# The Victimisation of V.A.D.s.

**Published:** 1919-09-20

**Authors:** J. E. Green


### THE EDITOR'S LETTER-BOX.

[Correspondence on all subjects is invited, but we cannot in any way be responsible for the opinions expressed by our correspondents, who must give their name and address as a guarantee of good faith, but not necessarily for publication. Correspondents are reminded that brevity of style and conciseness of statement greatly facilitate early insertion.]

### The Victimisation of V.A.D.s.

*To the Editor of* THE HOSPITAL.

SIR,—It is a great pleasure to see that THE HOSPITAL has taken up the question of the unjust treatment of a section of the V.A.D.s. I have served both at home and abroad since September 1914, and it seems to me almost unbelievable that while the nursing V.A.D.s get a full gratuity, the general service section—cooks, clerks, etc.—get nothing. Their work abroad was especially good, and at one hospital, the 43rd General, Salonica, I know that they almost entirely took over from the men, doing all the roughest work.

Till I went abroad, in August, 1916, I worked at a military hospital at Exeter. There many of the cooks came from domestic service, where they had been drawing good salaries from £40 to £50 a year. They gave up all this and were given by the War Office £26 a year. Surely these women are entitled to something after from four to five years' work?

Perhaps an even harder case is one of a V.A.D. who, at the beginning of the war, wished to be a nurse, but was requested by her Headquarters to become a clerk, owing to a great shortage in the last-named. Consequently, she can now draw no gratuity, although she has done a steady five years' work.

I am sure I am speaking for the majority of nursing V.A.D.s when I say that they consider the injustice done to their fellow-workers a grave one.—I am, yours truly,

J. E. GREEN, V.A.D.

